# Letter from Boston

**Published:** 1857-04

**Authors:** 


### LETTER FROM BOSTON.

BOSTON, December, 1856.

There are two medical journals in this city. Why, then, send one's thoughts abroad for publication? Well, there are things that one cannot say at home, you know. One has to be particularly tender in talking of home matters before folks; and then one's own people don't like to have you tell their deeds to them, be they good or bad.

MEDICAL JOURNALS IN BOSTON.

Two journals? Yes, we have two. The *Boston Medical & Surgical Journal*, which for many years was under the editorial management of Dr. J. V. C. Smith, changed hands a year or two since, and although he remained nominally editor, it was well understood here that Drs. Minot and Morland were, and were to be, the sole editors. It was said here that Clapp, the owner, was afraid to turn off his former editor, because he threatened to publish another journal at a less price, if he did. His courage, however, was at last raised to the proper point, and the change was made. The result of the change was the "*Medical World.*" I can't say how much of a hole it has made in the subscription list of its predecessor, but it is seldom seen on the tables of the profession in Boston. Homœopathy, hydropathy, everything which can be called collateral humbugs and collateral sciences, gets admission to its columns. Dr. Smith has the ability to edit a journal. He is an indefatigable industrious man; but having early in his professional career taken a public office, the usual fate of good-natured politicians awaited him. It is hard to be an independent politician. To be an independent medical editor and a politician at the same time is an impossibility. The *Medical World* probably has but very few subscribers in Massachusetts, and will not be a long-lived periodical, unless the homœopathists or the Female College take it up. There are enough of the former in the United States to back it, and enough women in trowsers who can be convinced of the immorality of men midwives, to subscribe for a publication which will support them.

The *Journal*, in the meanwhile, pursues the even tenor of its way. I fear that the editors are not sufficiently independent of the publisher to allow everything to go in that they could wish. The profession in Boston do not support them as it ought to. The older practitioners here seldom write, and the younger ones were so long kept in wholesome fear of their seniors that they are only beginning. The editors, however, are always courteous, and every one knows that they are able. Still, the *Journal* is rather wanting in the spice which should season a weekly paper.

### HOSPITALS.

There is talk of a new hospital, and the profession have prepared a petition to the city government, setting forth the necessity. There is actually, in this city of one hundred and seventy thousand people, not a single public bed for lying-in women! There is a building called the Lying-in Hospital, but its doors have been closed for some two or three months. Some say want of funds shut it up, others say that there was a quarrel between one of its medical staff (for it had three physicians for fifty patients a year) and the ladies who visited it, which caused the closing up. Be this as it may, the nearest free lying-in bed is three miles down the harbor; a most excellent distance, when it is considered that Boston harbor sometimes freezes for four miles down.

Lying-in women are not the only ones for whom a hospital is wanted. The Massachusetts General Hospital is very hard to gain admission to. There are almost always applications for the admission of chronic cases, waiting for weeks a chance for admission. You can always see phthisis there, but the acute fevers seldom, unless one is placed in a bed belonging to some private individual.

The Boston Dispensary is becoming one of the useful institutions. It is a private charitable association. Many of our own physicians are said to have obtained a start there. For several years past the anxiety to obtain the situations fell away, because of the miserable plan of its organization, and men were sought for the places in vain. Dr. Lawrence, son of the late Amos Lawrence, took the matter in hand last year, and it has been entirely re-organized. There is now a central office, and a corps of physicians and surgeons attached, who will not fail to make it all that it should be; and I am much mistaken if, in ten years from this time, it will not be considered quite as valuable to the profession and the public as any hospital in the country. I do not know all the gentlemen who now are attached to it, but the names of Williams, Lyman, Slade, Blake, Stone, and others well known in Boston, will become well known to the country in due time.

In addition to these, there is a class of ward physicians, younger men, who make visits to patients at their houses. The former receive no pay for their services, the latter have one hundred dollars a year each. The average income of a physician's first year is, ordinarily, no more than that. It will therefore be the means of encouragement to many a young man whose means are small, and whose determination to succeed is strong.

This does not make up for the want of a new hospital. The poor who can support themselves should be found the means of support; those who cannot, the public ought to take care of, and the sick poor are certainly of the latter class. The filling of our almshouses and hospitals with the lazy, has been the means of immense suffering to the worthy. It is not a disgrace to be poor. It ought not to be thought so, and if there was more attention paid by the public purse to the relief of misfortune, instead of leaving it to private charity, idleness would not so often receive the reward which should be given to those who are willing, but have not the means.

EYE AND EAR INFIRMARY.

The Eye and Ear Infirmary is another of our private establishments for the relief of distress. Why this and the Dispensary have not been taken up as a means of educating physicians long since, no one can tell. But we have no clinical institution in Boston, which amounts to a tithe of what Boston could produce. The result is that Boston students are running off to New York and Philadelphia for want of home education. Perhaps at a future day you will learn why medical schools do not flourish in this city, where all other educational projects succeed, and where the means are abundant, nothing being wanted but the proper management.

MEDICAL SOCIETIES.

The medical societies here are active in their way. There are three of them. First on the list is the Suffolk District Medical Society. I say *first*, because it is the society to which all the regular physicians belong. It is a branch of the State Society. Its officers are a President, who is *ex-officio* a Vice-President of the State Society, a Vice-President, Secretary, and Treasurer.

Meetings are held on the last Saturday evening of each month, at which papers are read, cases reported, and discussions had upon medical topics. The meetings are usually dull, because there are one or two gentlemen who occupy most of the time in matters peculiarly interesting to themselves. Once or twice within the last year, the hours have been occupied in listening to the complaints of a single member of the profession, who seems to have an idea that he is persecuted, although no one knows why, nor how.

Two or three times a year there are stated meetings for police business, elections, &c. They occasionally have tried members for mala praxis. There is no very great chance, however, of one's being convicted, the offenders bullying the members into not voting. Two members only have ever been expelled, so far as I can learn. One of these had already become known unfavorably to our courts, and the other made a defence too feeble to save him.

The principal medical transactions are by the other two societies, both somewhat aristocratic in their management. Their mode of electing members is not known to the profession at large. The older of these is the Boston Society for Medical Improvement. It has a large and exceedingly valuable pathological museum, collected mostly, I believe, by Dr. J. B. S. Jackson, whose fame as a pathologist is not confined to Massachusetts. They meet on the second and fourth Mondays in each month. What business they do can be easily seen in the *Boston Medical and Surgical Journal*. You will see by its columns that many of the older and more distinguished of the profession are on the roll of members.

The other society is the Society for Medical Observation. They say that this society is peculiar in its work. Papers are read by the members in turn, and dissected without much regard to the feelings of writers by the audience. Few men are willing to brave the criticisms to which they are likely to be exposed, and consequently applications are not likely to be numerous for membership. Still, no one who is not a member of either of these societies can tell much about them, except that they are both very hard to get into.

I forgot to mention that the latter society has a valuable library of journals, which are distributed among the members, and passed from hand to hand in regular order. Their bound volumes stand in the hall, which is occupied by the three societies, and of themselves are a standing inducement to medical men to join.

There is also in Boston a College of Pharmacy. When they meet I cannot tell, nor whether they do much. There is so much bad physic in the world, that the services of this college are needed. It is no difficult matter to become a member. The being a gentleman seems to be a sufficient qualification; for there are among them those who hardly have any other. But this association is young, and the formation of it shows that the druggists are alive to a sense of their own deficiencies.

At some future day, I may give a slight account of the druggists, and (I trust they will pardon me for speaking of them in the same sentence) of the quackery of Boston. STUDENT.

---

Boston, January 9, 1857.

HEALTH OF BOSTON.

Boston, for some reason or other, has not been a very profitable home for physicians for the past few years. Whether this is to be attributed to the increased supply of pure water, or whether there is a periodical increase of disease and a return to health, and that we have been at the return swing of the pendulum for a few years, I cannot say. It is evident, however, that Boston has enjoyed an immunity from disease, that is not likely to last.

SCARLET FEVER.

The daily papers will tell you that Scarlet fever has been making terrible ravages this season. This is true to a certain extent. So true is it, that if we should have as many cases of yellow fever as there have been deaths, in some weeks, from Scarlet fever, the city would be deserted by a very large proportion of its inhabitants. And yet the former, almost every physician feels confident, is non-contagious, the latter eminently contagious.

Scarlet fever has not been so violent, nor so common, as is generally imagined. There are physicians who have hardly seen one case a month during the last year, while in active and extensive practice. Others have been constantly occupied with the cure of it, their cases numbering, it is said, more than one hundred each. The cause of this difference is to be sought in the class of business. Through the summer, it is my impression, that the fever was confined to the lower Irish and German population. From the middle of July to the first of September, was the vacation of the public schools, and there was little intercourse between these and the American children. During these weeks, a large number of those children, whose parents are able to bear the expenses, are in the practice of leaving the city. From the first of October, the fever began to spread in the schools, and, as the season went on, it was more common among the children of American parents. The deaths have never been excessive among the latter. Greater cleanliness, better ventillation, and more careful attention to the prescriptions of medical attendants, are a sufficient explanation.

There is no prevaling system treatment of Scarlet fever in Boston. All the remedies possible, from mercurials to cold effusion, are in vague, including anointing, by one gentleman, with cold cream, and another with olive oil, and from this through the various greases, to bacon rind.

HOMŒOPATHY AND BELLADONNA.

The Homœopaths, during the fall and winter, have been harvesting. The dread of parents is so great, that they are willing to try any prophylactic, and many a child is supposed to owe its life to belladonna, who never was exposed to the disease. At last some of the apothecaries have caught the alarm, and fearful lest some other purses than their own should be filled, and willing to pander to the fears of parents, have displayed ounce vials filled with something which looks marvellously like distilled water, but is labelled "Belladonna." Why not?

The homœopaths have a way of calling everything scarlet fever, which is attended with redness of the skin, and even a sore throat, which has no such complication, passes for this disease. They are not all guilty of deception in this matter, for the greater number of homœopathic practitioners are totally unable to distinguish between different diseases. Did it ever occur to you, my dear Doctor, to count up the number of men who were able to get a living by regular practice, who had deserted it for homœopathy? Are they not usually the men who have failed in the practice of medicine, and adopted the practice of this peculiar form of quackery, on account of its gentility? Thomsonism, the quackery of the kitchen, requires too much labor, and some little knowledge. Homœopathy, the quackery of the parlor, requires no previous education, because, if honestly practiced, it never does any immediate mischief.

Did you ever know any thoroughly educated physician in your State, who, whatever his poverty might be, took up the practice? If you ever did, was he a man whose judgment or candor in other matters was at all to be relied on? I never heard of one.

This is rather aside from scarlet fever and belladonna. The Female Orphan Asylum in this city is said to have been sensibly afflicted during this season. Stories of numerous coffins carried in by night have horrified the timid, and horrid pictures in the air have been drawn by the tongues of the marvellous. The truth is, I learn from good authority, that one-fourth of the children, less than thirty, have had the disease. All of these had it mildly, but one, who died. What an opportunity of testing belladonna! Softly. Belladonna was tried upon every child in this same house, years ago, as a prophylactic. The number exposed was the same as this year. The number who took the fever was the same. The number of deaths was the same. Moreover, the epidemic was not so common outside the walls as this year.

### "PRIVATE AND CONFIDENTIAL." TRALL'S CIRCULAR.

While I was visiting, this evening, a circular was brought to my door, headed "Private and Confidential." Now, on Paddy's plan, that not being able to keep a secret, he would tell it to some one who could, I shall let you know what it is. One J. Silas Brown, "agent for Boston and vicinity," has requested me, *confidentially* of course, to act as his *deputy* agent, he being the agent of one R. T. Trall, M. D., (so says the principal circular,) of 15 Laight St., New York. I presume that Dr. Trall is one of the distinguished men of New York, although I never heard of him before. Of course that only "argues myself unknown." The object of the agency is primarily to collect statistics upon the production of sex at pleasure; secondarily and really, to inform the profession that he has discovered a method of preventing pregnancy. The circular is, in fact, one of those filthy advertisements, probably intended to fall into the hands of those who make a business of seduction, and a trade in female virtue. It would not be worth one's while to allude to this circular, but that certain physicians in Boston, having the desire to investigate the electro-chemical baths, rather patronized this J. Silas Brown, who keeps some of the tubs in Lagrange Place. As these gentlemen stand well in the profession, perhaps they may be pleased at being asked to act as his agents. The price of the precious knowledge is only $100, "one-half of which will be abated, on the presentation of satisfactory evidence that the party is really poor." There is no information as to whether clergymen are informed gratis; but, to make the thing take with the public, in other words, to make it cocksure, this should not have been omitted.

### THE INFLUENCE OF FASHION ON SURGERY.

We are in the midst of winter, and cold it is even for winter. The Metropolitan Railroad, which runs its horse cars through the main street of Boston, will doubtless have its effect on our profession. The inability of the drivers to turn out obliges the passers-by to keep to the right, as they should. There are few omnibuses to cross from side to side depositing passengers—consequently the streets are less often blocked, and accidents less frequent. Every one knows how the practice of surgery has been influenced, at times, by the fashion of dress. High side-pockets in the great coats were attended with fractures of the olecranon. When the pockets came down, so did the fractures, and the radius and ulna were the endangered bones. If, with the former, the humerus was oftener dislocated, with the latter the forearm bore the brunt of the blow. Why may not the introduction of horse railroads produce an entire revolution of fashion, in the style of accidents? Tight lacing, our elders tell us, produced deformity of chest and cough. Heavy skirts, tied about the body, released the chest to a certain extent, but the effect was seen in the abdominal organs, and various uterine displacements and discharges followed. Who can foresee the sequel of crinoline? Is it to be rheumatism, or neuralgia? dysuria or metritis?

How many cases of amaurosis will little bonnets produce? Or is iritis to be the fashion? Verily, civilized life has its ills.

FORTHCOMING WORKS BY DRS. DURKEE AND CHANNING.

It is announced that Dr. Durkee, of this city, is about publishing a work on venereal diseases. I believe he claims to have some new views to bring forward on the subject.

The Boston practitioners have not been before the public much, as book-makers. Two or three ex-professors in the Massachusetts Medical College have collected their papers from journals, and have given them to the world in duodecimos; but no treatises of any length have appeared. It is generally believed that Dr. Walter Channing has a work in hand, the result of some forty years' experience in his specialty. Whatever it may be, every one believes it will contain a large number of facts, and that at any rate it will be readable.

Yours truly, STUDENT.

